# Laparoscopic single port cystolithotomy using pneumovesicum

**DOI:** 10.1590/S1677-5538.IBJU.2015.0337

**Published:** 2016

**Authors:** Hoon Choi, Jae Hyun Bae

**Affiliations:** 1 Department of Urology, Korea University Ansan Hospital, Korea University College of Medicine, Korea

## Abstract

*Purpose:* Currently, several modalities are used to manage bladder stones. We report laparoscopic single port cystolithotomy using stone basket via pneumovesicum method.

## INTRODUCTION

Case description: Patient 63 years old, male with multiple bladder stones. Our cystolithotomy procedure was carried out under general anesthesia with dorsal lithotomy position. With rigid cystoscopic view, the bladder was maximally distended with saline solution, then a 10-mm laparoscopic trocar was placed transvesically at four centimeters in the midline above the pubic symphysis. Then, the optical transmission medium was changed from water to CO2 gas and we gained better operative vision after suction with irrigation. By use of CO2 gas within the bladder instead of saline irrigation, we can prevent the overflow of saline out onto the skin or extravesical tissues. Furthermore, air enables more clear view than water under the limited light source.

We performed the operation under a intravesical pressure of 10 to 12mm Hg. At first we tried to remove stones by forceps, but it could not be passed through the trocar. We used stone basket to grasp the stone one by one and eventually we could remove all the stones through trocar without additional use of laparoscopic specimen bag or other instruments. We closed the port site with a subcutaneous suture and bond application.

## RESULTS

The whole procedure was completed without any complication and there was no injury to bladder mucosa. The operative time was 42 minutes and blood loss was negligible. Total number of extracted stones was 16 and maximal diameter of biggest stone was 1.3cm. Foley removal was done in 3 days and the patient was discharged after spontaneous voiding in the 4th day the operation. Patient had no history of bladder outlet obstruction and did not complained of voiding symptoms. So no additional management was proposed.

## DISCUSSION AND CONCLUSIONS

Usually, these patients are treated with transurethral cystolithotomy using holmium laser or other energy. But any kind of fragmentation technique need the stone to be fragmented into small pieces to be irrigated out even with the use of holmium laser, or ultrasonic probe lithotripsy. Large prostates with serious intra-vesical protrusion or an elevated bladder neck can make the removal of fragments difficult with the risk of postoperative urethral strictures if manipulation is done via urethra. Also, the risk of bladder mucosal injury or perforation must be taken into account. So, the proposed technique has several advantages in relation to transurethral procedures. Also, it is possible to remove the Foley catheter earlier than when transurethral cystolithotomy is performed (3 versus 7 days).

Our procedure has some limitations. If stones are bigger than 15mm, our procedure may not be applied. Elbahnasy et al. were able to minimize this problem by using a self-retaining laparoscopic trocar (1). Others used an endo catch bag to capture the stones before lithotripsy. This made the procedure more efficient as fragments would be trapped into the bag during lithotripsy, so allowing for easy removal of bladder fragments from the bladder (2). And the bag provides a barrier between the lithotripter and the bladder wall providing a protective layer, which minimizes trauma during the procedure. Extracorporeal pneumatic lithotripsy or Kelly forceps using bag could be applied too, in case of large stone if our technique is not available (3).

## ARTICLE INFO

**Available at:** **www.intbrazjurol.com.br/video-section/Choi_1047_1048/**

**Int Braz J urol. 2016; 42 (video #11): 1047-8**
